# Supplementary material for: Assessment of the Antibiofilm Performance of Chitosan-Based Surfaces in Marine Environments
Source: Int J Mol Sci. 2022 Nov 24;23(23):14647. doi: 10.3390/ijms232314647 (PMC9741481; doi:10.3390/ijms232314647)
Supplement: Supplementary file 1 [file ijms-23-14647-s001.zip › ijms-2012473-supplementary.pdf]

Supplementary Materials

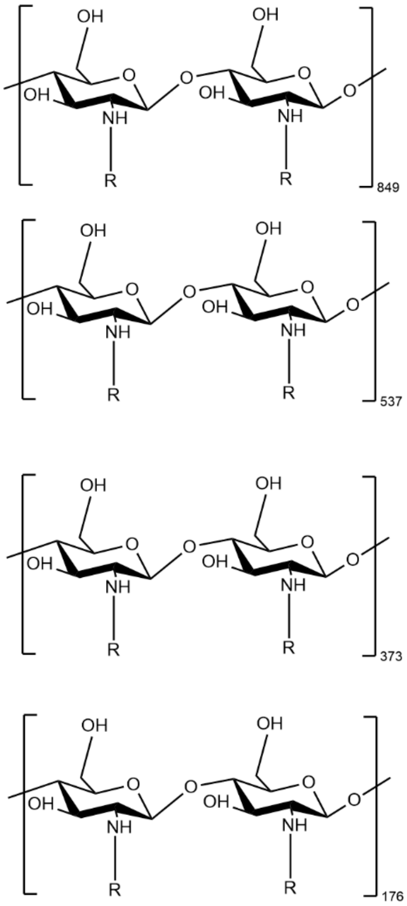

β-CS

CS1

CS2

CS3

R= H, CH_3CO

Figure S1. Chemical structures of the native chitosan (β-CS) and its three depolymerized derivatives (CS1, CS2, and CS3).
